# A dive into the bath: embedded 3D bioprinting of freeform *in vitro* models

**DOI:** 10.1039/d3bm00626c

**Published:** 2023-07-20

**Authors:** M. Özgen Öztürk-Öncel, Baltazar Hiram Leal-Martínez, Rosa F. Monteiro, Manuela E. Gomes, Rui M. A. Domingues

**Affiliations:** a 3B's Research Group I3Bs – Research Institute on Biomaterials, Biodegradables and Biomimetics, University of Minho, Headquarters of the European Institute of Excellence on Tissue Engineering and Regenerative Medicine, AvePark – Parque de Ciência e Tecnologia Zona Industrial da Gandra Barco Guimarães 4805-017 Portugal megomes@i3bs.uminho.pt rui.domingues@i3bs.uminho.pt; b ICVS/3B's – PT Government Associate Laboratory Braga/Guimarães Portugal

## Abstract

Designing functional, vascularized, human scale *in vitro* models with biomimetic architectures and multiple cell types is a highly promising strategy for both a better understanding of natural tissue/organ development stages to inspire regenerative medicine, and to test novel therapeutics on personalized microphysiological systems. Extrusion-based 3D bioprinting is an effective biofabrication technology to engineer living constructs with predefined geometries and cell patterns. However, bioprinting high-resolution multilayered structures with mechanically weak hydrogel bioinks is challenging. The advent of embedded 3D bioprinting systems in recent years offered new avenues to explore this technology for *in vitro* modeling. By providing a stable, cell-friendly and perfusable environment to hold the bioink during and after printing, it allows to recapitulate native tissues’ architecture and function in a well-controlled manner. Besides enabling freeform bioprinting of constructs with complex spatial organization, support baths can further provide functional housing systems for their long-term *in vitro* maintenance and screening. This minireview summarizes the recent advances in this field and discuss the enormous potential of embedded 3D bioprinting technologies as alternatives for the automated fabrication of more biomimetic *in vitro* models.

## Abstract 2

This minireview highlights recent advances on the application of embedded 3D bioprinting concepts for the fabrication of *in vitro* models.

## Graphical abstract

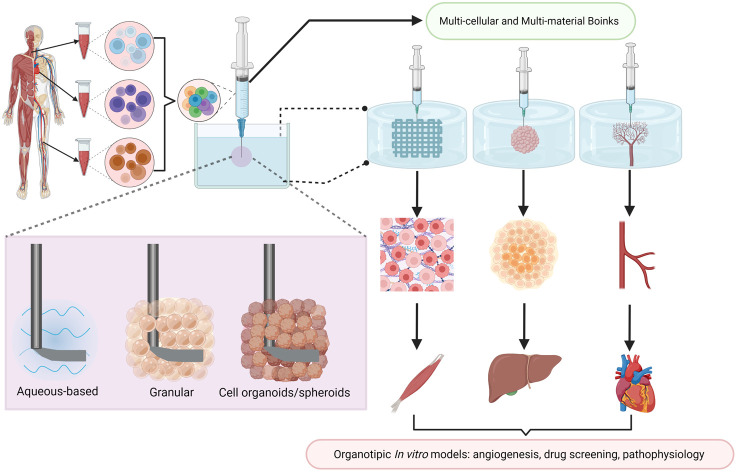

## Introduction

*In vitro* models are fundamental preclinical tools for (patho)physiological studies and drug discovery pipelines.^1^ Due to the growing evidences that typical 2D *in vitro* models developed on flat tissue culture plastic have limited predictive power on the *in vitro*–*in vivo* extrapolations, the last decade has seen a marked increase on the search for *in vitro* human microphysiological systems (MPS) that can recapitulate organ-level functions.

3D bioprinting is an evolving additive manufacturing technology, where materials with biological components are deposited in a layer-by-layer manner according to a predefined digital file to form a 3D product.^2^ According to ASTM standards,^2,3^ bioprinting techniques can be categorized into four main methods, *i.e.*, extrusion based,^4^ jetting based,^5^ laser based^6^ and vat photopolymerization based^7^ printing systems. Among the aforementioned methods, extrusion-based systems are the most widely dissiminated^8^ technique for the fabrication of cellularized constructs^9^ mimicking tissues/organs function for developing physiologically relevant 3D *in vitro* models, or for possible implementation as therapeutic options for the treatment of diseases.^10^ However, one of the main limitations of these 3D bioprinting systems^11^ is associated with the difficulties in bioprinting complex multilayer constructs, due to the inherent physical properties of bioinks typically based on soft hydrogel matrices, which, when printed, tend to get deformed or collapse.^12^ These unavoidable effects of gravity^13^ on hydrogels at the air–water interface, make the print of elaborate or intricate structures and maintenance of its fidelity a challenging task.^14,15^

To overcome these limitations, a surge of new methods involving the use of physical media that provides external support^16^ to the embedded bioinks while bioprinting have been developed in recent years, enabling the design and manufacture of 3D constructs that better resemble the biological structures of the human body. The use of support baths-assisted systems allows to print freeform constructs with lower geometric restrictions, higher resolution^17^ and smaller size features,^18^ a very important factor, for example, for bioprinting vascular-like structures,^19,20^ which are integral components of advanced dynamic MPS integrating tissue-like perfusion. Importantly, a key advantage of bioprinting in support baths is the possibility of using low viscosity bioinks,^21^ which favors the viability of printed cells and its general biological performance in printed constructs.^22–25^ Similarly, compared to traditional unsupported strategies, these systems further facilitate the bioprinting of multimaterial^26^ and multicellular structures.^27^ Moreover, since the crosslinking process takes place when the bioprinting of all the material is finished^28^ and not layer-by-layer as in conventional methods,^29^ an additional advantage of bioprinting in support baths is the possibility of accelerating the manufacturing process of cellularized constructs, opening the door for the manufacturing escalation^30,31^ of the bioprinting process, reducing printing times and costs.^32^

Together with the increased spreading and availability of extrusion-based bioprinters at most bioengineering facilities, all these factors contributed to make of embedded 3D bioprinting a trending technology which allows the fast and accurate fabrication of freeform constructs with complex geometries directly in support baths, which might have temporary or permanent structural functions.^33^ Recent innovations have further expanded the range of applications of this technology in the biomedical field. In the following sections we identify and discuss the outstanding potential of embedded bioprinting systems as options for the manufacturing of more precise *in vitro* models, discussing how the integration of other bioengineering technologies and tissue-specific bioink materials in its design concepts is opening new avenues to be explored in this particular field.

## Engineering support baths for embedded 3D bioprinting of *in vitro* models

**Fig. 1:**
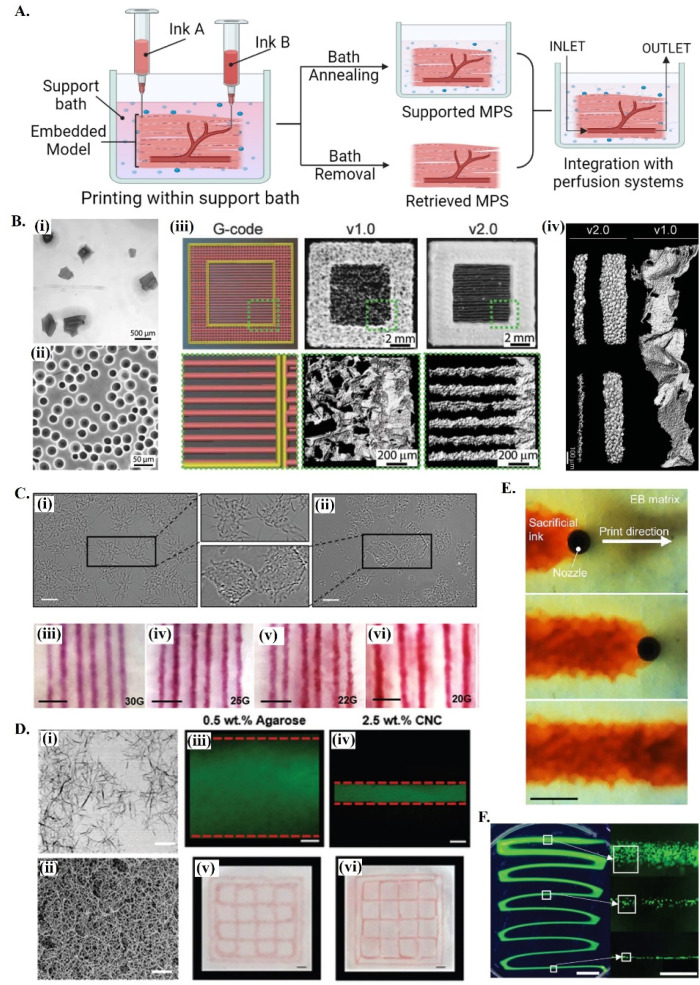
Printing in different support baths. (A) A schematic drawing on embedded 3D bioprinting steps followed by bath annealing to generate a supported MPS or bath removal to retrieve the 3D bioprinted structure. Fabricated microphysiological system can be further integrated with perfusion systems for dynamic culture. (B) 3D bioprinting of collagen filaments using FRESH v1.0 and v2.0. (i) Large and irregularly shaped gelatin microparticles in the support bath v1.0 and (ii) spherical and relatively smaller ones in FRESH v2.0. (iii) Comparison of printed constructs obtained using the same G-code. (iv) 3D bioprinted single collagen filaments in FRESH v1.0 and in FRESH v2.0, showing the increase in the resolution and the shape fidelity by improving the bath properties. Reprinted with permission from ref. 51. Copyright 2019 American Association for the Advancement of Science (AAAS). (C) 3D bioprinting in agarose support bath. (i) Micrographs of agarose fluid gels showing small subunits and (ii) agarose slurry with angular particles. (iii–vi) Printing resolution in agarose fluid bath using needles with various diameters. Scale bars: 5 mm. Reprinted from ref. 36 under the terms and conditions of the Creative Commons CC BY 4.0 License. (D) 3D bioprinting in cellulose nanocrystals (CNCs) support bath. (i) Rod-shaped colloidal form and (ii) self-assembled fibrillar structure of CNCs and comparison of printing resolution (scale bar: 200 nm) in (iii and v) agarose (scale bar: 250 μm) and in (iv and vi) CNCs (scale bar: 1 mm). Reprinted with permission from ref. 38. Copyright 2021 John Wiley and Sons. (E) Time-lapse images showing 3D printing of sacrificial gelatin ink (red) within living OBB support matrix. Scale bar: 1 mm. Reprinted from ref. 43, under the terms and conditions of the Creative Commons CC BY 4.0 License. (F) Low viscosity ink 3D printing within ATPS. Fluorescent images of thinning filaments (left, scale bar: 5 mm) fabricated with ATPS, providing high resolution cell patterning (right, scale bar: 500 μm). Reprinted from ref. 52 under the terms and conditions of the Creative Commons CC BY 4.0 License.

Reproducing the natural complexities of tissue microenvironment with biomimetic *in vitro* models will enable a better understanding of tissue regeneration stages, in addition to disease progression and the outcomes of potential treatment options. In the development of bioengineered MPS by extrusion-based 3D bioprinting, support baths offer significant advantages stemming from their ability to hold the deposited bioink during and after bioprinting or, if needed, the printed structure can be released by the removal of the bath^34^ (see Fig. 1A). The implementation of these concepts strongly depends on the properties of the support bath.

Typically, these fluid media are made of stress-yielding materials with self-healing properties. At resting state, these viscoelastic materials show a solid-like behavior. When the stress exerted by nozzle movement is higher than the yield stress of the support bath, the material undergoes localized solid–liquid transition around the nozzle. The initial support bath solid state is recovered when the stress-induce deformation is removed, keeping the printed structures suspended in its position, thus enabling the high-resolution printing of complex 3D structures.^46^ This self-recovery property facilitates bioprinting with a wide range of hydrogel options as bioinks, and eventually enhance both the bioactivity and the biomimetic architecture of printed structures.^47^ Although the required features of support baths vary depending on the target application, they should present adjustable rheological characteristics, biocompatibility and provide long term cell culture and/or easy removal.^48^ For further insights on the specific rheological requirements of support baths, readers are referred to detailed reviews on this topic.^49,50^

Support baths can be synthesized from different fluid biomaterials, ranging from polymer hydrogels to living spheroid/organoids (see Table 1). Among all types, granular hydrogels have been extensively used in embedding printing due to their easy production methods, in addition to its self-recovery and shear-thinning properties.^53^ Their rheological behavior can be easily tuned by engineering the physical and chemical nature of forming microparticles, their packing densities or by using mixed types and sizes of microparticles.^53–55^ One of the most explored support baths for producing *in vitro* models is the well-known freeform reversible embedding of suspended hydrogels (FRESH) system (Fig. 1A and B), which is originally based on gelatin microparticles.^56^ In the first version (FRESH v1.0), gelatin microparticles with an average diameter of around 65 μm were obtained by simple mechanical blending [Fig. 1B(i)]. However, the regularity and resolution of printed filaments using FRESH v1.0 was relatively limited due to the large and highly variable shapes of the microparticles [Fig. 1B(iii) and (iv)]. These printing parameters were significantly improved by the second generation FRESH v2.0 [Fig. 1B(iii) and (iv)], where spherical gelatin microparticles with reduced particle size and polydispersity are produced by coacervation methods [Fig. 1B(ii)].^51^ In general, FRESH shows yield stress behavior, allows freeform bioprinting and its liquid compartment is compatible with many bioinks’ (*e.g.*, alginate, collagen, dECM, fibrinogen, HA) crosslinking mechanisms.^51,57^ Once the printed structure is cured, it is released from the surrounding bath by simply melting gelatin at 37 °C. Besides gelatin, various other granular hydrogels have been used as support baths, including agarose (Fig. 1C),^36^ alginate,^20^ gellan gum,^58^ xanthan gum^59^ and Carbopol-based^60,61^ ones.^62^ For instance, agarose particulate slurries are shear-thinning and self-healing fluids with fast recovery rates well adapted to embedded bioprinting systems, although the resolution of obtained prints is lower compared to *e.g.*, the gelatin microparticle-based bath of FRESH v2.0. Bulk hydrogel based on reversible physical crosslinkings, such as host–guest HA^63^ or xanthan gum^59^-based hydrogels, also show the required shear-thinning pseudo-plasticity to be used as support baths and its continuous matrix ensure improved printing resolution compared to granular systems. Depending on the chemical nature and physical properties of the bath, the resolution, size and 3D architecture of produced tissue models can be adjusted,^51^ and other support bath removal strategies (*e.g.* enzymatic cleavage or mechanical separation) can be employed. Detailed discussion on existing hydrogel systems and its crosslinking mechanisms being explored for bath production can be found in recent dedicated reviews.^33,62^

**Table 1:** Various support bath types and inks for the embedded bioprinting of *in vitro* models

Bath type	Bath material	Ink	Application	Ref.
Granular	Alginate microparticles in xanthan gum-suppl. growth medium	Decellularized omentum and sacrificial gelatin	Vascularized heart model	20
	κ-Carrageenan (CarGrow)	Fibrin	Bone-like, cardiac-like constructs	35
	Agarose fluid gel	Collagen, gellan gum, alginate, and i-Carrageenan	Carotid artery, T7 invertebral disc	36
	Alginate microparticles in-collagen & laminin & fibronectin & hyaluronic acid (HA)	Stem cells & sacrificial gelatin	Neural models, vascular-like channels	25
	Carbopol	GelMA	*In vitro* neuroblastoma model	37
Nanoparticle-based	CNCs	Gelatin, GelMA, alginate, platelet lysate, Pluronic F-127, tendon dECM	Tumor-on-a-chip model, *in vitro* tendon models	38–40
ATPS	Oxidize bacterial cellulose	Poly-l-lysine	*In vitro* vessel model	41
	Poly(ethylene oxide)	Poly(acrylic acid)-dextran	On-demand *in vitro* tissue models	42
Organoids	iPSC derived OBBs	Sacrificial gelatin	Perfusable cardiac tissues	43
Decellularized extracellular matrix (dECM)	Skin derived dECM	Vascular tissue-derived dECM	*In vitro* melanoma model	44
	Vascular tissue derived dECM	Calcium-Pluronic F127	*In vitro* atherosclerotic model	45

*In vitro* maturation is crucial for creating functional MPS, allowing printed cells to secrete new ECM and establish essential connections with their microenvironment.^34^ However, maintenance of the complex architecture of printed cellular constructs over long cultivation times can be challenging, particularly when using ECM-based bioinks (*e.g.*, collagen, fibrin or decellularized ECM) or high cell density constructs, where deformation (contraction) or disruptions of printed constructs may easily occur. This concern tends to increase as the dimension of printed structures decreases, thus being particularly relevant for miniaturized MPS. An emerging biofabrication strategy to overcome these limitations levering on the many functionalities of support baths is the “print-and-grow” concept, where the supporting media can be maintained or annealed post-printing to provide structural support for long term culture of printed constructs. These strategies have been implemented using different cell-friendly support materials, such as κ-Carrageenan,^35^ CNCs^38^ or modified HA.^64^ The liquid component of the support baths can also include selected ECM components, such as collagen, fibronectin, HA or laminin, to improve the biomimicry and adjust the functionality of the system. Annealing of these composite materials for “locking” the structure after high fidelity bioprinting generates a stable and cell-interactive matrix for long term functional development of target tissues models.^25^ This concept allows to provide functional housing devices or living environments, the typical microfluidic bioreactor of organs-on-chip, to the printed constructs for their long-term maturation/maintenance and screening. Several methods can be applied for annealing the bath depend on its nature,^62^ including *e.g.*, thermally induced crosslinking of ECM proteins exiting in its composition,^25^ enzymatic crosslinking of gelatin-based hydrogels (*e.g.* by microbial transglutaminase),^65^ or by promoting nanoparticles self-assembly with addition of biocompatible ions (*e.g.* Ca^2+^ for CNCs) (Fig. 1D).^38^ Characteristics of these systems such as cell microenvironment mimetic micro- and nano-features, permeability to cell nutrients/metabolites, structural stability and transparency make them promising platforms for the automated arraying of physiologically relevant *in vitro* models.^35,38,64^

Besides providing physical support for the bioprinting process and housing the fabricated models for their *in vitro* maturation and screening, the bath can also incorporate living components where other cellular structures can be printed. This bioprinting strategy allows to simultaneously replicate both the external and internal components of a target organ by bioprinting *e.g.*, blood vessels *via* sacrificial inks within the support bath incorporating the stromal tissue cell.^65^ This technique has been effectively applied on the fabrication of a complex vascularized ventricle *in vitro* model with a fast, unparalleled bioprinting ability, which is not easy to carry out using standard bioprinting technologies.^66^

Aqueous two-phase systems (ATPS) are an innovative class of active support baths which have just started to be explored in recent years. Aqueous-based support platforms consist of bioink–immiscible liquid environment that function as a supportive fluid and/or pregel during bioprinting of complex microstructures, where both phases remain at equilibrium until solidification.^41,52^ Formation of hydrogen bonding between bioinks and support liquid provides noncovalent interactions, allowing these liquid architectures to be stabilized through different mechanisms, such as interfacial complexation^42^ or other biocompatible crosslinking mechanisms.^52^ Low viscosities and the relative interfacial tension of bioink and matrix solutions enable high-speed bioprinting without compromising cell viability. Moreover, exceptionally low fiber diameters can be reached (Fig. 1F)^52^ and bioprinted interconnected, cell-lined channels can be fabricated in one-step bioprinting aproaches.^41^ Facile handling of these complex freeform architectures suspended in a liquid phase or the possibility of its locking within a crosslinked hydrogel matrix presents valuable opportunities to be explored in the fields of tissue modeling, organs-on-chip and tissue engineering for the precise and more practical fabrication of arbitrary vascularized constructs with compartmentalized phases.

## Combining embedded 3D bioprinting with other biomanufacturing technologies

**Fig. 2:**
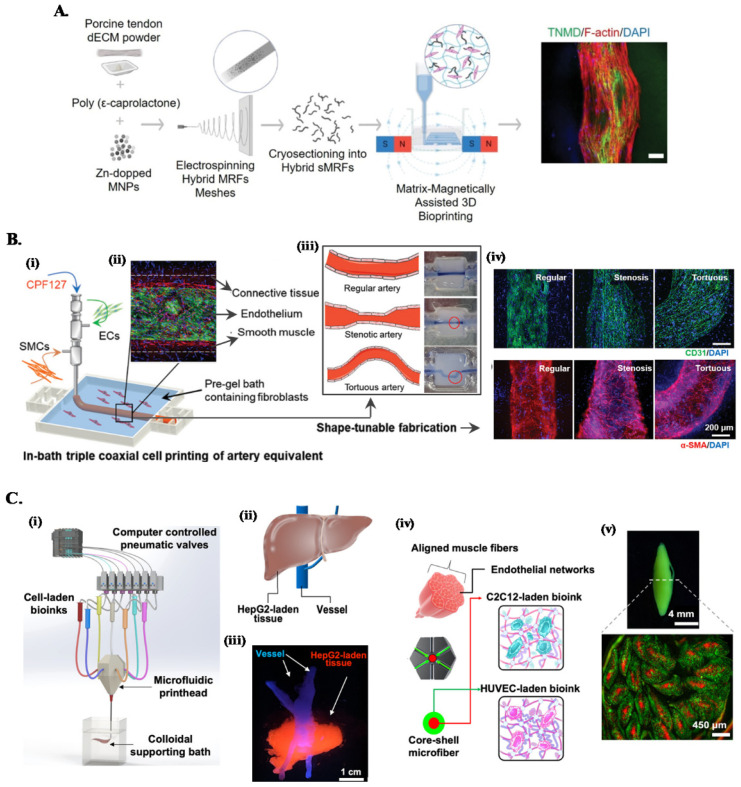
Combined embedded 3D bioprinting approaches for engineering *in vitro* tissue models. (A) Magnetically and dECM assisted embedded 3D bioprinting of tendon biomimetic composites. Flowchart of the process, showing the production of magnetically-responsive microfibers made of tendon dECM, PCL and Zn-doped magnetic nanoparticles, magnetically assisted 3D bioprinted in CNC support bath and the obtained tendon mimetic construct, stained for tenomodulin (green), cytoskeleton (red) and nucleus (blue). Scale bar: 250 μm. Reprinted with permission from ref. 40. Copyright 2022 John Wiley and Sons. (B) Embedded triple coaxial bioprinting of triple layered atherosclerotic *in vitro* model. (i) Experimental design for the generation of in bath bioprinting with (ii) printed artery equivalent. (iii) Shape-tunable fabrication schematics, showing that tunable arterial construct designs can be achieved by adjusting the printing speed and path. (iv) Maturation of generated blood vessels mimetic structures with endothelium (in green) and smooth muscle cells (in red). Reprinted with permission from ref. 45. Copyright 2020 John Wiley and Sons. (C) Microfluidic, multimaterial, embedded 3D bioprinting to fabricate compartmentalized constructs. (i) Schematic of the 3D printing system showing the major components. (ii) Design and (iii) cellular print of liver-mimetic construct with vessels (in blue) and hepatocellular carcinoma cells (HepG2)-laden bioprinted tissue (in red). (iv) Core–shell design and (v) printed muscle fiber-like construct with c2c12 myocytes (in red) in the core and HUVECs (in green) in the shell. Reprinted with permission from ref. 68. Copyright 2022 American Chemical Society.

Mimicking the complex architecture of native tissues, composed of multiple cell types and molecules organized into specific cell patterns within confined volumes, has been a major fabrication challenge of functional *in vitro* models. The convergence of conventional embedded bioprinting systems with different advances in biomanufacturing technologies (see Fig. 2) is being leveraged as strategy to tackle this challenge.^45,67^

One approach is combining customized multichannel housing devices made of biocompatible hydrogels pre-printed in support baths, with subsequent channel cell-lining and incorporation of tumor spheroids to build reproducible vascularized *in vitro* neuroblastoma models.^37^ To recreate the anisotropic organization of cells and ECM of tissues such as tendons or muscles, we have developed a magnetically-assisted embedded bioprinting system allowing to control the alignment of magnetically-responsive microfibers incorporated in bioinks, which guide cell growth and organization and can be further used for the remote stimulation of cells after bioprinting (Fig. 2A).^40^ However, typical extrusion printheads with single nozzle can just print single bioink struts, limiting the achievable axial complexity of printed structures. The combination of these systems with co- and triaxial nozzles widens significantly the design space that can be explored on the biomanufacturing of *in vitro* models, as these extruders enable to produce filaments layered with different bioinks according to the design of the different nozzle compartments. For instance, triple coaxial nozzle has been applied to print three-layer vascular structures with adjustable geometries and dimensions (Fig. 2B). In-bath bioprinting of these constructs with irregular shapes and multiple vascular cell types enabled to mimic the specific signaling events in atherosclerosis, enabling this system to be used as a potential *in vitro* atherosclerotic model for the evaluation of therapeutic molecules.^45^

On the other hand, combining microfluidics-based printheads with embedded bioprinting platforms further improves the spatial complexities of compartmentalized, multicellular, microfibrous constructs that can be built. This approach has been applied to print liver- and muscle fiber-mimetic constructs with perfusable vessels, where a multimaterial microfluidic printhead with a single nozzle provided fast switching between various bioinks, eliminating the alignment concerns during nozzle switching (Fig. 2C).^68^ Moreover, ECM-like colloidal gel-based support bath offered a microenvironment to enhance the spatial organization of bioinks, printing fidelity and speed to build complex constructs.^68^

Current fabrication strategies of organ-mimetic constructs for therapeutic applications have shown limited success due to the challenges in the recapitulation of human scale, complex microarchitectures with densely packed, multiple cell types of functional native tissues.^69,70^ Considering that the main aim of bioengineered *in vitro* models is to recreate key functional hallmarks of human tissues and organs, the same limitations apply to these systems. The merge of organoid and embedded bioprinting technologies might provide a possible solution to overcome this challenge.

**Fig. 3:**
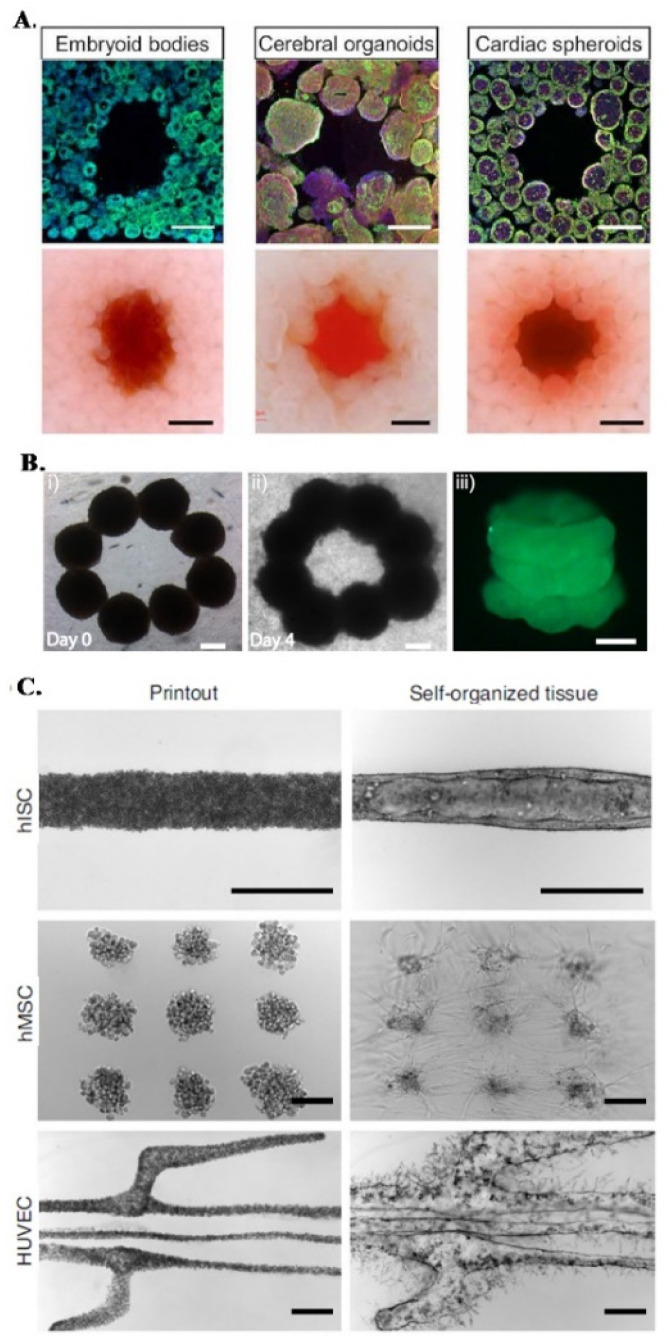
Embedded bioprinting combined with organoid technology. (A) Sacrificial writing into OBB matrices, composed of embryoid bodies, cerebral organoids and cardiac spheroids. Cross sections of matrices showing the sacrificial printing with (row 1) immunostaining of indicated OBBs and (row 2) their brightfield images. Scale bars: 500 μm. Reprinted from ref. 43, under the terms and conditions of the Creative Commons CC BY 4.0 License. (B) 3D bioprinting of microtissue models through spheroids. Directed fusion of bioprinted ring-shaped spheroids into microtissue rings on (i) day 1 and (ii) day 4 of culture. (iii) 3 layers of fused spheroids after their removal from the support bath. Scale bar: 200 μm. Reprinted from ref. 64 under the terms and conditions of the Creative Commons CC BY 4.0 License. (C) Bioprinting-assisted tissue emergence (BATE) approach with self-organizing, organoid forming cells. Using BATE for the patterning of various cell types [from top to bottom: human intestinal stem cells (hISC), human mesenchymal/progenitor cells (hMSC), human umbilical vein endothelial cells (HUVEC)] (i) right after bioprinting and (ii) after self-organization. Scale bars: 500 μm. Reprinted with permission from ref. 70. Copyright 2020 Springer Nature.

Organoids have been recently proposed as building blocks for the production of physiologically relevant constructs,^43,62,68,69^ as they show unique self-organization potential and specific tissue mimetic features.^70^ For instance, sacrificial bioprinting in a support matrix made of organ building blocks (OBBs) produced from induced pluripotent stem cell derived (iPSC) organoids, creates scalable, perfusable tissue mimetic constructs with vascular networks. OBBs contain high cell densities and exhibit self-healing and viscoelastic behavior, supporting 3D freeform bioprinting of single or branched channels *via* sacrificial inks (Fig. 1E and 3A).^43^ Furthermore, diameters and resolution of these perfusable channels can be changed by tailoring OOB properties, *i.e.*, characteristic diameter.^43^ Recent advances on the development of anisotropic OBBs might further expand the potential range of applications of this biofabrication strategy, as demonstrated for bioinks made of anisotropic OBBs that allowed to print functional aligned cardiac microfilaments with enhanced contractile performance.^43,64,70,71^

In a different approach, Daly *et al.* achieved higher resolutions by bioprinting high cell density spheroids into a self-healing support media with shear-thinning properties. The non-adhesive and viscoelastic nature of this supporting HA-based hydrogel enabled controlled fusion of spheroids to form stable microtissues with predefined architectures and high cell viabilities (Fig. 3B).^64^ An interesting demonstration of organoid-integrated bioprinting potential is a study by M. Lutolf group where stem cells and organoids were directly printed into Matrigel and collagen mixture support matrices (Fig. 3C).^70^ Embedded 3D bioprinting technology was adopted to guide tissue morphogenesis, providing these self-organizing cells or cell aggregates a defined tissue mimetic shape and spatial arrangement, allowing to obtain interconnected, multicellular constructs. With this organoid fusion concept, constructs with more physiologically relevant scale can be produced and various supportive cells can be used to adjust the self-organization and remodeling features of organoids.^70^

Biofabrication of *in vitro* models also requires the use of biomaterials that recreate the specific cellular niche of the target tissue or organ.^72^ Numerous hydrogel matrices such as alginate,^73,74^ agarose,^75^ HA,^76,77^ chitosan,^78,79^ gelatin,^80,81^ collagen,^82,83^ silk^84,85^ or gelatin methacryloyl (GelMA)^86,87^ have been proposed to formulate bioink. However, their potential to closely mimic the rich tissue-specific cell microenvironment is limited.^8^ In the past few years, dECM hydrogels have emerged as a promising alternative for bioink formulation in *in vitro* modeling applications, as dECM retain the main biochemical and biophysical cues of the respective niches, exhibiting superior biofunctionality when compared with other available hydrogel options.^88^ dECM hydrogels can be produced by controlled decellularization and digestion of tissues obtained from different organs (*e.g.* brain,^89^ colon,^90^ tendon,^39,91^ bone^92^ and heart^93^). In terms of composition, these biomaterials are a rich source of collagens, glycoproteins, growth factors and other important components that are crucial to dictate cell behavior, function, and fate modulation.^94,95^ The superior bioactivity of dECM-based hydrogels in comparison to other materials has been widely demonstrated and their combination with embedded bioprinting concepts enables to overcome the inherent rheological and structural limitations of these biomaterials as bioink hydrogels. For instance, a recent study compared the cellular performance of gelatin and kidney dECM bioinks using agarose as support bath for the bioprinting process.^96^ In this study, the presence of renal specific markers could only be detected on the dECM constructs and, while in the presence of kidney dECM cells were able to establish a confluent and highly interconnected network, gelatin-encapsulated cells remained round-shaped, with absence of evident network formation.^97^ The wide biofabrication potential provided by combining dECM bioinks with embedded bioprinting concepts has been leveraged for engineering more complex multicellular systems. For example, we have recently explored our CNC support bath platform to 3D write multicellular tendon models using tendon dECM bioinks, where the printed tendon stroma and vascular compartments enabled to study the cellular crosstalk established in these MPS.^39^ In this type of multicellular systems, coaxial printheads are particularly interesting options because it allows compartmentalization of printed bioinks in the same strut. The versatility of this approach was demonstrated in the fabrication of functional volumetric vascularized muscle tissues, where skeletal muscle and vascular dECM bioinks were coaxially printed on granular gelatin support baths.^98^ Besides assisting the bioprinting process, 3D printed muscles showed enhanced alignment of the matured myotubes *in vitro* and increased vascularization and innervation *in vivo*.^98^ Interestingly, dECM can be used not only as bioink hydrogel but also as the actual cell-laden stress yielding support baths, which can be gelled post-printing.^44,45^ This is a particularly appealing strategy for *in vitro* modeling because bioink and support baths can be formulated with different dECM hydrogels to retain the specific signatures of different functional biological structures of its tissue of origin.^47^ For instance, in a recent study, skin derived dECM bath was used for the embedded bioprinting of 3D cancer models incorporating perfusable blood and lymphatic vessels printed with vascular tissue-derived dECM.^44^ These models allowed the biomimetic recapitulation of metastatic steps of melanoma and were used to screen the different inhibitor combinations of drugs to suppress its metastasis. As another example, a multicellular atherosclerotic *in vitro* model incorporating endothelial, smooth muscle and connective tissue cells was developed leveraging on this concept.^45^ The proposed coaxial cell bioprinting system used vascular tissue-derived dECM as cell-laden and functional support bath, and was explored to fabricate stable and perfusable three-layered conduits with tunable geometry, allowing to study both co-cultured cells and local turbulent flow signaling *in vitro*.

## Outlook and future perspectives

The fast increase in the number of studies applying embedded bioprinting for *in vitro* modeling in recent years demonstrates the outstanding potential of this technology in the field. It enables not only the high throughput and automated replication of multicellular MPS with arbitrary geometries emulating native tissues architecture and cellular patterns, but it also simultaneously allows to build these organotypic 3D constructs within their own tailor-made housing support for dynamic *in vitro* maturation and screening. All these capabilities position embedded bioprinting as a technology with a biomanufacturing potential difficult to be matched by other current *in vitro* modeling alternatives. However, this potential is just starting to be unblocked.

Looking toward the future, 3D bioprinting in support baths has the possibility of adopting emerging technologies such as machine learning^99,100^ and artificial intelligence that allow faster modeling and simulation^60^ of the rheological, chemical and biological properties of the baths to adapt them to the new bioinks that are in development,^101,102^ thus allowing to manufacture *in vitro* models with better mechanical properties, better printing resolution and allowing to improve cell viability during and after the bioprinting process.^103^ One challenge to consider is the development of support baths with greater optical transparency,^38,104^ particularly if they are aimed to be annealed and used as functional housing device of printed constructs. This will enable better visualization of the resolution and fidelity of printed construct, as well as minimize light scatter effects that negatively affect the observation of MPS using standard microscopy techniques. On the other hand, the effect of bath removing strategy and its residues remaining on the bioprinted constructs on cell functions are still unclear. Ensuring cell survival by either developing new approaches to eliminate them or using the residues (or the annealed bath itself) to add new functionalities to printed constructs (*e.g.*, as carrier systems for release of bioactive molecules) is an underexplored but important issue to consider.^105^

The possibility of using support baths not only during the bioprinting process but as a continuous medium^35^ with modulable properties over time^106^ (4D bioprinting) or the controlled release of growth factors^107^ or genetic material^108^ is another alternative that is certainly worth to be explored. The same applies to the use of sacrificial inks with programmable dissolution rates.^109^ This strategy will allow temporal control over the spatial compartmentalization of the different cell population, which have different differentiation and/or maturation requirements that need to be considered before allowing their direct physical contact on the support “bioreactor” device. On the other hand, ATPS will enable to explore the fabrication of tubular structures to model different types of vascular diseases, and to embed perfusable microfluidic structures within the printed models with higher resolution, without concerns of extrusion speed neither ink viscosity, or requirement for removal of sacrificial inks.^41,52^ Despite performing better than most non-supported extrusion-based bioprinting techniques, another general challenge where the current in-bath 3D bioprinting technology still face some difficulties is in the fabrication of physiologically-relevant human-scale models capable of capturing the multiscale hierarchical complexity of native tissue in a fast and reproducible manner. The recent integration of embedded extrusion with volumetric printing concepts might be a promising strategy to mitigate these issues, allowing the fast production of large-sized sample replicates with increased cellular and architectural complexicity.^110^

Capability for real-time assessment of physiological event is an additional desirable functionality in advanced *in vitro* models.^111^ Integration of microelectronic sensors^112–115^ allowing real-time measurement of *e.g.*, changes in pH,^116^ temperature, or mechanical properties^117^ in the fabricated models will enable the *in vitro* monitoring of cell fate or other physiological signals of interest in these MPS. In this particular aspect, integration of machine learning concepts will certainly play key roles in the future of MPS for processing multimodal data inputs in a unified manner.

These are a few examples of the potential directions to be explored for evolving the current state of the art in this field. However, the possibilities are certainly much wider. Therefore, we foresee exciting developments being made in the coming years by integrating embedded bioprinting with new concepts and technologies.

## Conflicts of interest

There are no conflicts to declare.

## Supplementary Material
